# Retraction Note: An evaluation of the potential consequences of drilling titanium and tantalum implants during surgery - a pilot study

**DOI:** 10.1186/s12891-019-2824-5

**Published:** 2019-10-24

**Authors:** Paweł Skowronek, Paweł Olszewski, Wojciech Święszkowski, Marcin Sibiński, Marek Synder, Michał Polguj

**Affiliations:** 1 Clinic of Orthopedic and Traumatology, Regional Hospital and Kochanowski Medical University, Kielce, Poland; 2 Faculty of Materials Science and Engineering, Warsaw University of Technology, Warsaw, Poland; 3 Clinic of Orthopedics and Pediatric Orthopedics, Medical University of Lodz, Lodz, Poland; 4 Department of Angiology, Medical University of Łódź, ul. Narutowicza 60, 90-136 Łódź, Poland

**Retraction Note: BMC Musculoskelet Disord**

**https://doi.org/10.1186/s12891-017-1784-x**

The authors have retracted this article [1] because it constitutes redundant publication [2]. The authors signed the licensing agreement for publication in *HIP International* before submitting the manuscript to *BMC Musculoskeletal Disorders*. All authors agree to this retraction.
